# Application and optimization of reference change values for Delta Checks in clinical laboratory

**DOI:** 10.1002/jcla.23550

**Published:** 2020-08-30

**Authors:** Jinyoung Hong, Eun‐Jung Cho, Hyun‐Ki Kim, Woochang Lee, Sail Chun, Won‐Ki Min

**Affiliations:** ^1^ Department of Laboratory Medicine Asan Medical Center, University of Ulsan College of Medicine Seoul Korea; ^2^ Department of Laboratory Medicine Hallym University Dongtan Sacred Heart Hospital, Hallym University College of Medicine Hwaseong‐si Korea; ^3^ Department of Laboratory Medicine Ulsan University Hospital, University of Ulsan College of Medicine Ulsan Korea

**Keywords:** autoverification, delta check, quality control, reference change value, uncertainty

## Abstract

**Background:**

Delta check is a patient‐based QC tool for detecting errors by comparing current and previous test results of patient. Reference change value (RCV) is adopted in guidelines as method for delta check, but the performance is not verified. We applied RCV‐based delta check method to patients' data and modified for application.

**Materials and methods:**

Reference change value were calculated using results of internal QC materials and biological variation data. Test results of 17 analytes in inpatients, outpatients, and health examination recipients were collected. The detection rates of currently used delta check method and those of RCV‐based method were compared, and the methods were modified.

**Results:**

Reference change value‐based method had higher detection rates compared to conventional method. Applied modifications reduced detection rates. Removing the pairs of results within reference interval reduced detection rates (0.42% ~ 10.92%). When RCV was divided by time interval, the detection rates were similar to prior rates in outpatients (0.19% ~ 1.34%). Using RCV multiplied by twice the upper limit of reference value as cutoff reduced the detection rate (0.07% ~ 1.58%).

**Conclusions:**

Reference change value is a robust criterion for delta check and included in clinical laboratory practice guideline. However, RCV‐based method generates high detection rates which increase workload. It needs modification for use in clinical laboratories.

## Graphical abstract

Reference change value (RCV) can be used for determination of delta check limit. However, this study revealed that simple adaptation of RCV for delta check limit can produce excessive detection and increase the laboratory's workload. Further consideration and modification are needed for RCV and delta check before actually applying them to the clinical laboratory.

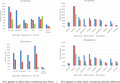

## 1 INTRODUCTION

Delta check, first proposed during the 1970s,^1^ is a patient‐based quality control tool. Delta is defined as the difference between current test results and prior test results. If a delta value exceeds the established limit, the test result is held for manual review by laboratory personnel^2^ such as considering the possibility of specimen mix‐up, checking the quality control data, examining the specimen quality, volume, and integrity, and reviewing the medical records of patient. At the beginning, delta check was frequently used as a tool for detecting laboratory errors including specimen mix‐ups. However, due to the introduction of the barcode system, the development of automatic analyzers, and other technological advances, delta check has a low positive predictive value for specimen mix‐ups.^3^ Nowadays, delta check seems to be more useful to become aware of clinically significant changes in the patient status.

Commonly used delta check methods include delta difference, delta percent change, rate difference, and rate percent change. Delta difference is defined as the absolute difference between current results and previous results. Delta percent change is defined as the percentage of the difference between the current results and the previous results divided by the previous one. Rate difference is defined as the delta difference divided by the interval between the current and past testing time, and the rate percent change is defined as the delta percent change divided by the interval between the current and the previous testing time. The selection of the delta check method depends on the extent of the test value, the distribution of the test value, etc.^4^ There is no consensus regarding specific methods such as which analyte should be applied to the delta check and which cutoff should be set. Currently, each medical institution has different rules for delta check.

There are two methods to determine the limit of delta check, that is, using biological variation and using patient data. CLSI EP33 “Use of Delta Checks in the Medical Laboratory” provides a detailed description of how to use the Reference Change Value (RCV) as a method of delta check limit determination using biological variation. RCV is the minimum difference of a measurement from a reference value that is considered as distinguishable from measurement uncertainty,^5^ and biological variation and analytical imprecision are used to calculate it. Measurement uncertainty is an expression of the statistical variance of a value due to a measured quantity.^6^ No measurement method is exact and when a quantity is measured, the value depends on various factors, such as the measurement system, the measurement method, the proficiency level of the measurer, and the environment. Even if an amount is measured several times in the same manner and situation, the measured value will differ from one measurement to another.^7^ An analytical imprecision can be obtained by reflecting the concept of uncertainty, and several attempts have been made to apply RCV calculated from this and the previously known biological variation data to delta check,^8^, ^9^ but which are not satisfactory yet. In this study, the delta check limit determined by using RCV is applied to the actual patients' data, and some modifications were tried toward the RCV‐based delta check method.

## 2 MATERIALS AND METHODS

### 2.1 Collecting data

We collected results of internal quality control materials (Lyphochek Unassayed Chemistry Control; Bio‐Rad Laboratories) over 1 year (2016) to calculate RCV. During 2016, QC materials with the same lot number were used. Test results of inpatients, outpatients, and general health examination recipients were collected over a 4‐week period (August 1‐28, 2018). The patients' previous test results within 60 days for outpatients and 40 days for inpatients were also collected and paired. Test results of the following analytes were collected: calcium (CA); glucose (GLU); creatinine (CREA); uric acid (UA); cholesterol (CHOL); protein (PROT); albumin (ALB); aspartate aminotransferase (AST); alanine aminotransferase (ALT); alkaline phosphatase (ALP); total bilirubin (TBIL); phosphorus (PHO); blood urea nitrogen (BUN); direct bilirubin (DBIL); sodium (NA); potassium (K); and chloride (CL).

Patient specimens and QC materials were tested using automated chemistry analyzers. A dedicated calibrator and a dedicated reagent provided by the manufacturer of each type of equipment were used. Roche Cobas C‐8000 (Roche diagnostics) was used for inpatient and health screening recipient samples, and Beckman Coulter AU5800 (Beckman Coulter) was used for outpatient samples.

### 2.2 Calculating RCV

Based on CLSI EP29‐A, the standard deviation of the inspection values of the internal quality control material for 1 year was used to calculate the uncertainty using the top‐down approach.^5^ Unacceptable internal QC data were not excluded. Two kinds of QC materials—high level and low level—were used, and uncertainty was calculated for each of the two automatic analyzers, thus yielding four uncertainty values per analyte. The largest of the four uncertainty values calculated was used as analytical imprecision (CVa) of each analyte. Within‐subject biological variation (CVi) was obtained from the Westgard Biological Variation database.^10^ Combined uncertainty was calculated using the calculated CVa and CVi values. RCV was calculated using the following formula.

$$\text{RCV}=k\times \sqrt{2}\times \text{combined uncertainty}=k\times \sqrt{2}\times \sqrt{\left({\text{CVa}}^{2}+{\text{CVi}}^{2}\right)}.$$

For coverage factor *k*, two types of Confidence interval (CI) (95% and 99%) were used to calculate RCV (*k* = 1.96 for the 95% confidence level and *k* = 2.58 for the 99% confidence level).

### 2.3 Applying the RCV‐based delta check method

RCV with 95% CI and RCV with 99% CI were used to compare the percent difference between the previous test and the current test results and used as the cutoff value of the delta percent change. For each analyte, the detection rate of the current delta check criteria^11^, ^12^ of our laboratory and the detection rate of the RCV‐based delta check method were compared. Results of the tests were compared separately for groups of Inpatients, outpatients, and health examination recipients based on the latest examination.

### 2.4 Modifying the RCV‐based delta check method

In the delta check rule setting process using RCV, some modification was performed in order to reduce the excessive detection rate. First, we excluded pairs of test results if both test results constituting the pair were within the reference interval of the analyte. The detection rate at this time was compared with the detection rate before the modification. Second, for analytes whose current delta check rule uses the rate difference and rate percentage change, the percent change was compared with the RCV with a 99% CI value divided by the interval of the test date, and the detection rate was calculated and compared with the detection rate before modification. As a third method, in the case of an analyte whose current delta check method uses the delta difference and rate difference, RCV with a 99% CI value multiplied by twice the upper normal limit value of the reference range was compared with the absolute difference of each pair and the detection rate was calculated and compared with those before the modification.

## 3 RESULTS

### 3.1 Data collection

Overall, 491 699 pairs of test results from inpatients, 332 840 pairs of test results from outpatients, and 32 100 pairs of test results from general health examination recipients were collected. A total of 66 842 test results of quality control materials were used to calculate RCV (Table 1). Current delta check methods and cutoffs in our hospital are shown in Table 2.

**Table 1:** Number of test results of quality control materials used for calculating the reference change values of each instrument and analyte

Analyte	Cobas C‐8000		AU 5800	
	Level 1	Level 2	Level 1	Level 2
Calcium	1379	1363	545	546
Glucose	1357	1351	656	648
Creatinine	1494	1504	581	574
Uric acid	1535	1532	557	554
Cholesterol	1549	1532	553	553
Protein	1456	1447	524	524
Albumin	1428	1437	557	556
AST	1241	1248	622	616
ALT	1212	1211	543	541
ALP	1281	1275	554	555
Total bilirubin	1252	1249	521	522
Phosphorus	1537	1537	530	530
BUN	1816	1820	560	560
Direct bilirubin	1241	1239	606	607
Sodium	1412	1405	529	528
Potassium	1371	1366	526	526
Chloride	1403	1392	534	532

Abbreviations: ALP, alkaline phosphatase; ALT, alanine aminotransferase; AST, aspartate aminotransferase; BUN, blood urea nitrogen.

**Table 2:** Current delta check methods and cutoffs in our hospital

Analytes	Reference interval	Inpatients			Outpatients		
		Currently used method	Currently used delta check cutoff		Currently used method	Currently used delta check cutoff	
			Upper	Lower		Upper	Lower
Calcium	8.6 ~ 10.2 mg/dL	DD	1.3 mg/dL	−1.3 mg/dL	DD	1.4 mg/dL	−1.4 mg/dL
Glucose	70 ~ 99 mg/dL	DD	132 mg/dL	−138 mg/dL	DD	145 mg/dL	−152 mg/dL
Creatinine	0.70 ~ 1.40 mg/dL	DPC	50%	−35.87%	DPC	55%	−39%
Uric acid	3 ~ 7 mg/dL	RD	0.83 mg/(dL × d)	−1.17 mg/(dL × d)	RD	0.9 mg/(dL × d)	−1.3 mg/(dL × d)
Cholesterol	0 ~ 199 mg/dL	RD	18 mg/(dL × d)	−20 mg/(dL × d)	RD	20 mg/(dL × d)	−22 mg/(dL × d)
Protein	6 ~ 8 g/dL	DD	1.3 g/dL	−1.5 g/dL	DD	1.5 g/dL	−1.7 g/dL
Albumin	3.5 ~ 5.2 g/dL	DD	0.8 g/dL	−0.9 g/dL	DD	0.9 g/dL	−1.0 g/dL
AST	0 ~ 40 U/L	DPC	277.78%	−70.27%	DPC	305%	−77%
ALT	0 ~ 40 U/L	RPC	82%/d	−24.36%/d	RPC	90%/d	−27%/d
ALP	40 ~ 120 U/L	RPC	80%/d	−40%/d	DPC	88%	−44%
Total bilirubin	0.2 ~ 1.2 mg/dL	DPC	200%	−66.67%	DPC	220%	−73%
Phosphorus	2.5 ~ 4.5 mg/dL	DD	2.1 mg/dL	−2.2 mg/dL	DD	2.3 mg/dL	−2.4 mg/dL
BUN	10 ~ 26 mg/dL	RPC	53.85%/d	−26.92%/d	RPC	59%/d	−30%/d
Direct bilirubin	0 ~ 0.5 mg/dL	RPC	71.43%/d	−28.57%/d	RPC	78%/d	−32%/d
Sodium	135 ~ 145 mEq/L	DD	7 mEq/L	−8 mEq/L	DD	8 mEq/L	−9 mEq/L
Potassium	3.5 ~ 5.1 mEq/L	DD	1.2 mEq/L	−1.2 mEq/L	DD	1.4 mEq/L	−1.4 mEq/L
Chloride	98 ~ 110 mEq/L	DD	8 mEq/L	−9 mEq/L	DD	9 mEq/L	−10 mEq/L

Abbreviations: ALP, alkaline phosphatase; ALT, alanine aminotransferase; AST, aspartate aminotransferase; BUN, blood urea nitrogen; DD, delta difference; DPC, delta change percent; RD, rate difference; RPC, rate percent change.

### 3.2 RCV calculation

Reference change value was calculated using CVa and CVi, and RCV with a 99% CI showed a significant difference from 4.4% to 136% depending on the analyte (Table 3). CVa did not vary significantly according to the analyte (1.05 ~ 7.42), but CVi showed a large fluctuation depending on the analyte, that is, 0.60 ~ 36.80. For analytes with CVi smaller than 5.0%, such as calcium, protein, albumin, sodium, potassium, and chloride, the RCV was smaller than 20%. And for analytes with a CVi larger than 12.2%, such as AST, ALT, total bilirubin, and direct bilirubin, showed a large RCV of more than 50%.

**Table 3:** Calculation of reference change values with 95% and 99% confidence intervals using CVi and CVa for each analyte

Analytes	CVi (%)	CVa (%)	RCV with 95% CI (%)	RCV with 99% CI (%)
Calcium	2.10	1.92	7.88	10.37
Glucose	5.60	2.23	16.70	22.00
Creatinine	5.95	4.59	20.84	27.44
Uric acid	8.60	2.07	24.52	32.29
Cholesterol	5.95	1.95	17.35	22.85
Protein	2.75	1.96	9.36	12.33
Albumin	3.20	2.65	11.52	15.17
AST	12.30	7.42	39.82	52.43
ALT	19.40	5.59	55.96	73.69
ALP	6.45	5.25	23.04	30.34
Total bilirubin	21.80	2.24	60.75	79.99
Phosphorus	8.15	4.44	25.73	33.88
BUN	12.10	3.03	34.58	45.53
Direct bilirubin	36.80	5.83	103.28	136.00
Sodium	0.60	1.05	3.35	4.41
Potassium	4.60	1.40	13.33	17.55
Chloride	1.20	1.51	5.34	7.03

Abbreviations: ALP, alkaline phosphatase; ALT, alanine aminotransferase; AST, aspartate aminotransferase; BUN, blood urea nitrogen; CI, confidence interval; CVa, analytical imprecision; CVi, within‐subject biological variation; RCV, reference change value.

### 3.3 Application of the RCV‐based delta check method to patients' data

In terms of the detection rate, both methods using 95% CI and 99% CI tend to have a higher detection rate compared to those using traditional cutoff (Table 4). This trend was more pronounced in outpatients than in inpatients. In exception, in patients with ALT and direct bilirubin, the detection rate was reduced when RCV was applied.

**Table 4:** Comparison of the delta check detection rate using the current delta check rule, RCV‐based delta check method with 95% CI, 99% CI, and 99% CI excluding pairs with both test results within the reference interval (RI) in inpatients (A), outpatients (B), and general health examination recipients (C)

(A) Inpatients				
Analyte	Detection rate (%)			
	Current	RCV‐based with 95% CI	RCV‐based with 99% CI	RCV‐based with 99% CI excluding within‐RI pairs
Calcium	2.37	13.22	7.14	6.47
Glucose	2.46	45.67	35.32	34.90
Creatinine	2.08	15.89	8.21	7.08
Uric acid	10.89	24.81	15.79	11.95
Cholesterol	8.69	21.38	13.18	2.25
Protein	2.67	25.22	14.94	11.19
Albumin	2.16	23.76	13.04	12.27
AST	1.95	18.69	12.03	8.32
ALT	12.54	8.98	6.22	4.04
ALP	0.86	12.33	6.99	6.10
Total bilirubin	1.18	10.12	6.43	2.46
Phosphorus	1.74	20.34	11.95	9.87
BUN	10.83	16.38	16.49	11.76
Direct bilirubin	13.99	3.48	2.88	1.92
Sodium	0.92	9.09	3.48	2.70
Potassium	1.81	17.98	9.27	5.17
Chloride	0.85	6.25	2.39	1.78

(B) Outpatients				
Analyte	Detection rate (%)			
	Current	RCV‐based with 95% CI	RCV‐based with 99% CI	RCV‐based with 99% CI excluding within‐RI pairs
Calcium	0.47	8.26	3.25	2.64
Glucose	1.74	35.55	26.56	26.03
Creatinine	0.78	9.84	4.74	3.66
Uric acid	0.27	19.19	11.75	7.04
Cholesterol	0.21	21.68	13.58	5.38
Protein	4.19	21.97	15.24	8.27
Albumin	1.71	17.05	10.90	9.86
AST	1.13	16.47	8.88	6.59
ALT	0.19	17.17	10.39	5.84
ALP	1.84	16.27	9.79	8.09
Total bilirubin	0.81	10.61	5.98	1.49
Phosphorus	0.90	14.85	8.57	6.14
BUN	1.34	26.36	26.45	15.36
Direct bilirubin	0.15	1.70	1.64	0.87
Sodium	0.97	10.52	4.52	3.47
Potassium	0.84	17.97	9.15	4.08
Chloride	1.89	11.77	5.85	3.68

(C) General health examination recipients			
Analyte	Detection rate (%)		
	RCV‐based with 95% CI	RCV‐based with 99% CI	RCV‐based with 99% CI excluding within‐RI pairs
Calcium	8.19	3.96	2.33
Glucose	11.86	6.72	6.26
Creatinine	9.00	3.95	2.89
Uric acid	10.66	4.75	2.19
Cholesterol	18.11	11.63	7.55
Protein	10.92	3.95	2.00
Albumin	7.15	2.97	1.30
AST	19.65	12.02	6.06
ALT	19.60	11.74	5.09
ALP	13.58	8.00	5.26
Total bilirubin	10.78	6.27	1.35
Phosphorus	10.21	4.07	1.86
BUN	17.56	8.00	4.43
Direct bilirubin	14.29	14.29	0.00
Sodium	8.25	1.99	0.76
Potassium	21.75	11.33	2.56

Abbreviations: ALP, alkaline phosphatase; ALT, alanine aminotransferase; AST, aspartate aminotransferase; BUN, blood urea nitrogen; CI, confidence interval; RCV, reference change value; RI, reference interval.

### 3.4 Modification of the RCV‐based delta check method

We tried adjusting RCV‐based delta check methods in several ways to find a way to mitigate over‐detection. First, we detected pairs exceeding the RCV with a 99% CI value, except when both of the results constituting the pair were within the reference interval (Table 4). In this case, the detection rate was lower than 10% in the group of health examination recipients. The detection rate decreased by more than 50% in cholesterol and total bilirubin for inpatients and in cholesterol, total bilirubin, and potassium for outpatients.

**Figure 1:**
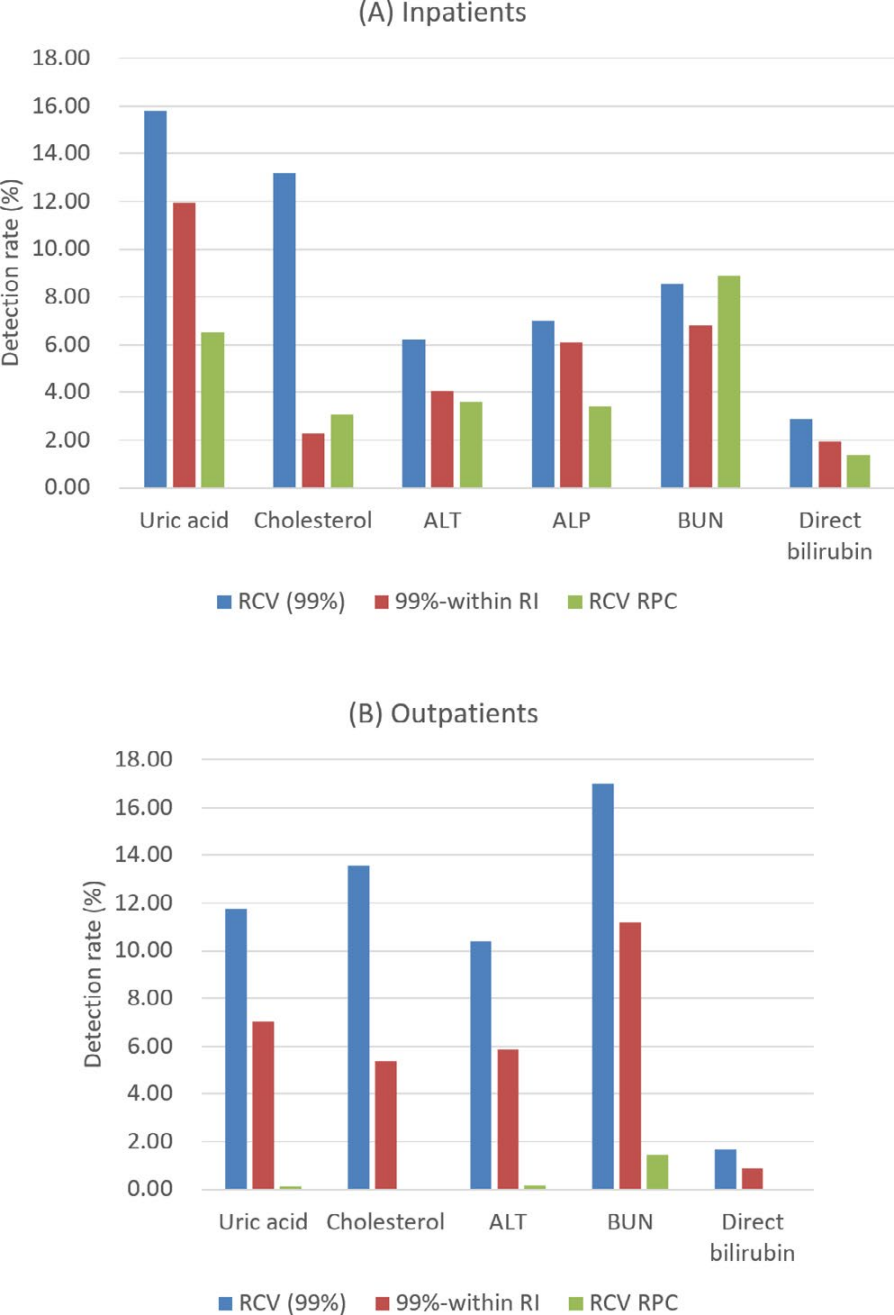
Comparison of the delta check detection rate (%) using RCV with 99% CI, RCV with 99% CI excluding within‐RI pairs, RCV with 99% CI divided by the interval of test date (RCV RPC) in inpatients (A) and outpatients (B). CI, confidence interval; RCV, reference change value; RI, reference interval

Second, if the existing delta check method has a rate difference or rate percent change, the RCV is compared with the rate percent change to reflect the time factor for the analyte (Figure 1). Applying this method, the detection rate in the outpatient group was 0.00 ~ 1.44%, similar to the conventional detection rate. In the inpatient group, except for ALP, all of them showed a reduced detection rate.

**Figure 2:**
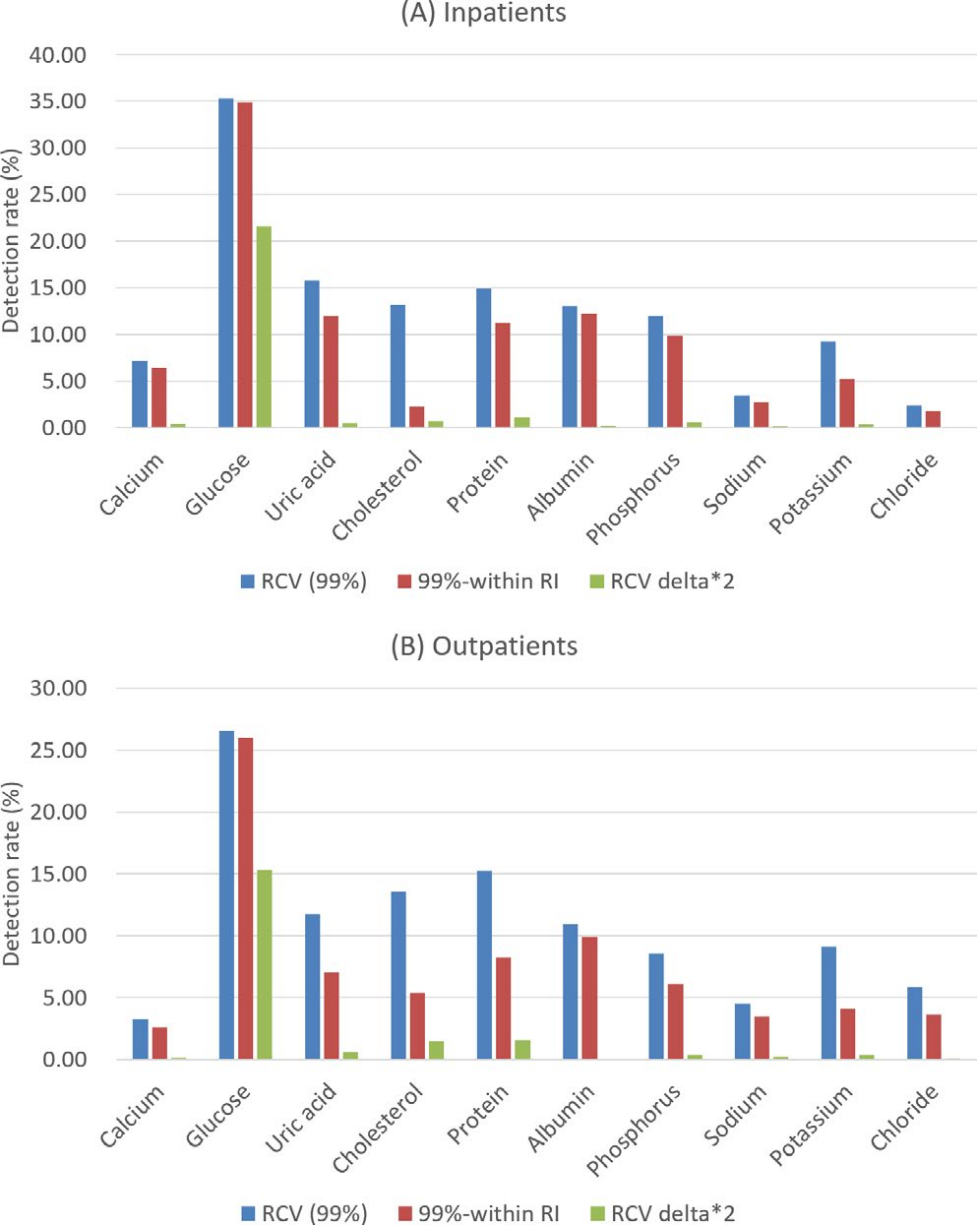
Comparison of the delta check detection rate using RCV with 99% CI, RCV with 99% CI excluding within‐RI pairs, and RCV with 99% CI multiplied by twice the upper normal limit in inpatients (A) and outpatients (B). CI, confidence interval; RCV, reference change value; RI, reference interval

As the third modification method, when the current delta check method uses a delta difference or rate difference, the value obtained by multiplying the RCV value by twice the upper limit of the reference value was used as the delta check cutoff. As a result of this method, all of the analytes except for glucose showed much lower detection rate of less than 2% in both the inpatient and outpatient groups (Figure 2).

## 4 DISCUSSION

The concept of the delta check was originally proposed by Nosanchuk and Gottman^13^ in 1974 as a quality control technique designed to find misidentified specimens. It was then established by Ladenson^1^ to compare the current and previous patient test results using the computer. The basic principle of this delta check has not changed much over the last 40 years. The delta check method using multivariate has also been attempted,^14^ but it is not widely used because of the computational complexity and burden on the laboratory information system.

The concept of uncertainty has been widely used in metrology and industry in many areas of measurement and engineering.^7^ In 2008, the Joint Committee for Guides in Metrology (JCGM) working group 1, expression of uncertainty in measurement, published Guide to the Expression of Uncertainty in Measurement (GUM). This uncertainty concept was introduced in the field of laboratory medicine, and the calculation of uncertainty and the concept of RCV are introduced in CLSI EP29‐A Expression of Measurement Uncertainty in Laboratory Medicine.^5^ The CLSI EP33 Use of Delta Checks in the Medical Laboratory also describes the use of RCVs for delta check cutoff settings.^15^ Studies using RCV when setting up the delta check rule were recently published.^8^, ^9^ In our study, as the simple application of RCV without modification showed over‐detection in all the analytes, it was considered that it would be difficult to apply RCV to delta check methods in clinical laboratories without modification.

Delta check is the best tool currently available for detecting specimen misidentification.^15^ However, the introduction of the barcode system and the development of automated specimen processing can reduce the utility of the delta check in detecting the specimen mix‐up in the current laboratory setting. Therefore, it is important to properly optimize between over‐detection and sensitivity because excessive false‐positive delta check alerts can cause delay in the work process and the reporting time of the laboratory. In the CAP Q‐Probe study, three delta check alerts were generated per 1000 tests in the median laboratory.^16^ With the present method of our laboratory, it is difficult to regard the current rule as appropriate because there were 39 delta check alerts per 1000 tests in hospitalized patients and 12 delta check alerts per 1000 tests in outpatients and thus generating more laboratory workload.

In calculating RCV, we first decided on the confidence interval. In this study, RCV was calculated using 99% confidence interval. The confidence interval of 95% for setting the delta check cutoff in the clinical laboratory tended to produce higher detection rate. The over‐detection tendency did not disappear even with the 99% confidence interval, suggesting that an additional modification was required for the RCV‐based delta check method.

Among the additional modifications, there was a method to exclude the pair from the delta check alert if both values were within the reference interval. The rationale for coming up with this method is that the main purpose of the delta check is detecting the laboratory error, but it can be also used to detect the change in a patient's condition. This modification can increase the probability that the delta check alert has actual clinical significance, and this method has actually contributed to the reduction of over‐detection in analytes such as cholesterol and total bilirubin.

The time factor is not reflected in the calculation formula of RCV. However, in the clinical laboratory, the rate which reflects time factor is often used in the delta check rule. In the case of an analyte that maintains a constant concentration in the body, such as sodium or chloride, the interval between test points has no significant impact on delta assessment. However, some enzymes, such as AST, are susceptible to false‐positive delta check alerts if changes over time are not considered, especially for outpatients since as the interval between two measurements is relatively longer than that of hospitalized patients, over‐detection occurs if the time factor is not reflected. Therefore, in the case of an analyte in which the time factor can play a critical role in the delta check, modification is necessary to reflect the time factor even if the cutoff is set using RCV.

In the case of an analyte with a narrow reference interval and a small change in the test result, if a percent change is used for a delta check, a small change may appear as a large value and generate over‐detection. Because RCV is basically a percentage value, universal RCV application cannot account for these characteristics of all the test items. Therefore, in this study, by using a formula multiplied by RCV and twice the upper limit value of the reference interval, we attempted to convert the RCV value, which is a percentage, to compare with the absolute difference, and we observed that it was of great help in reducing over‐detection.

On the other hand, in the current delta check rule used by our laboratory, positive delta and negative delta are set differently in analyte such as creatinine, AST, ALT, ALP, total bilirubin, BUN, and direct bilirubin. In Ko's study, the positive delta is larger than the negative delta in the analyte that reflects tissue damage and in the analyte that can rise due to a patient's condition.^8^ Considering this aspect, though it is not dealt with in this study, when RCV is applied, both limits are set equal, and it should be considered whether this is a proper delta check rule.

In glucose, the detection rate of 10% or less was not achieved in any of the delta check rules used in this study. This may be due to the clinical characteristics of the analyte. Glucose has a very large variation during a single day, depending on diet or blood sugar depressants or insulin administration. Therefore, it is difficult to regard a large change in glucose as a laboratory error such as specimen mix‐up. Of the total 46 institutions in the Q‐Probe study conducted by CAP, 16 used glucose as a delta check analyte and which is less than half of the total.^17^ When choosing analytes that should undergo delta check, analytes with frequent measurement and high individuality are likely to have advantage in detecting specimen misidentification. Index of individuality is the ratio of within‐subject biological variation to between‐subject biological variation, and when the index of individuality is below 0.60, the analyte is considered as having high individuality.^15^ Taking this into consideration, a comprehensive review of the adequacy of the delta check rule using RCV as well as which analyte is appropriate for laboratory error detection is required.

In conclusion, we applied the delta check rule reflecting RCV to the actual clinical laboratory test results and proposed modified delta check rule, which corrected and supplemented the shortcomings of RCV only method. Clinical laboratory managers need to review the delta check that has been done on a daily basis comprehensively from applicable items to detailed application methods. It is necessary to understand the concept and basic principle of RCV and to use it to set delta check rule and cutoff to balance between the laboratory workload and the laboratory error detection.
